# A database of schemes that prioritize sites and species based on their conservation value: focusing business on biodiversity

**DOI:** 10.1186/1472-6785-7-10

**Published:** 2007-09-27

**Authors:** Arthur G Blundell, Tormod V Burkey

**Affiliations:** 1 Biodiversity Neutral Initiative, 2102-1238 Melville Street, Vancouver, BC, V6E 4N2, Canada; 2 DNV Research, Veritasveien 1, N-1322 Høvik, Norway; 3 Current- Welhavensgate 19, N-0350 Oslo, Norway

## Abstract

**Background:**

Biodiversity offsets are conservation projects used mainly by business to counterbalance the environmental impacts of their operations, with the aim of achieving a net neutral or even beneficial outcome for biodiversity. Companies considering offsets need to know: (1) if there are areas of such biological importance that no impact is acceptable, and outside of these *no-go areas*, (2) the relative importance of biodiversity in the impacted site versus the site(s) proposed for protection, to ensure that the offset is of equal or greater status than that lost through the company's operations. We compiled a database of 40 schemes that use various methods to assess conservation priorities, and we examined if the schemes would allow companies to answer the above questions.

**Description:**

Overall, schemes tend to be designed to guide conservation organizations in their own priority setting or they categorize species based on conservation status. Generally, the schemes do not provide all the necessary information for offsets because they operate at a broad spatial scale or with low spatial resolution, which make it difficult to assess sites at the project level. Furthermore, most schemes do not explicitly incorporate *threat*, which we consider key to assessing whether offsets protect habitats or species that would otherwise be lost (*i.e*., provide *additionality*). The schemes are useful, however, for identifying the major conservation issues in different ecosystems around the globe.

**Conclusion:**

Companies can proceed by first avoiding, reducing, and mitigating impacts, and then using existing schemes to identify i) no-go areas and ii) appropriate offsets to compensate for any unavoidable loss in biodiversity. If existing schemes are inadequate, then companies should use integrated conservation planning techniques to define offset options within the region of their operations.

## Background

As a fundamental component of risk management, leading companies now go beyond regulatory requirements to mitigate a broad range of environmental and social impacts. This helps maintain their social license to operate. However, corporate responsibility is poorly developed in the area of biodiversity, especially in developing countries where biodiversity is greatest [1]. This lack of action contrasts strongly with the plight of the world's biodiversity, which most recognize as highly threatened [2].

According to the voluntary guidelines of the Convention on Biodiversity, companies should first avoid, reduce, and then mitigate their impacts on biodiversity. One tool being developed to deal with the residual impacts is the *biodiversity offset*, wherein a company finances conservation project(s) that compensate for the unavoidable impacts of its operations, so there is no overall negative effect on biodiversity [3,4]. In some countries, offsets have become standard practice. For example, in Western Australia, government approval for new projects requires "net conservation benefits" that go beyond typical project-level mitigation [3-5]. These biodiversity offsets are similar to carbon offsets, wherein a company sequesters an equivalent amount of carbon to that it emits, thus ensuring that the company is 'carbon neutral' [6]. Compared to greenhouse gases however, biodiversity offsets have even greater complications that concern measurement, stakeholder preferences, and equivalence [3,4]; Table 1 summarizes the basic steps in an offset process. This paper examines if existing schemes that assess conservation priorities can be used to determine whether the habitat or species protected by an offset is at least equivalent in status to that affected by a company's operations, *i.e., equivalence*.

**Table 1:** Process for developing a biodiversity offset (adapted from [4])

(1) Determine the baseline conditions with respect to biodiversity at the company's site
(2) Assess the likely impacts of the project
(3) Identify potential offset options consistent with conservation priorities
(4) Assess the baseline conditions at the offset site(s) and determine if they are commensurate with the predicted losses caused by the company's impacts
(5) Determine an appropriate offset replacement ratio (*i.e*., offset units per unit of impact)
(6) Develop a plan for managing and monitoring the offset and the company's project (to ensure project impacts do not diverge from estimates and expectations)
(7) Obtain legal and financial assurances to secure tenure of the offset site, support long-term management and monitoring, and cover remedial actions in the event of failure
(8) Implement the offset in accordance with plans and "best practices."

Stakeholder involvement will be key (and most likely decisive) in all stages of an adequate offset process.

In the context of biodiversity, schemes that assess conservation priorities serve two major purposes in determining 'status'. In addition to determining equivalence, the schemes should help identify no-go areas, *i.e*., areas deemed too important, or where habitat loss is already too great, to allow any further impacts. Such a safeguard is necessary to avoid the further loss of rare and irreplaceable habitats and species. For example, companies in the International Council on Mining and Metals have committed themselves to avoid mining or exploration in World Heritage Sites [7]. A failure to manage biodiversity impacts has led to successful market campaigns against prominent companies [8].

## Construction and contents

We compiled a database of schemes whose overall aim is to identify sites or species of conservation concern. We recorded the scale and spatial resolution at which these schemes operate, their specific objectives, and link to their websites. Redford et al. [9] make the distinction between schemes that assess 'where to do conservation' and the various approaches on 'how to do conservation'. Here, we focus on the issue of conservation priorities – the 'where'. Techniques – the 'how' – provide the actual methodology used to develop schemes. However, we do not review these individual methodologies here. The purpose of this paper is to compile a database of schemes that can be used by companies to ascertain the conservation status of the sites where they operate and to identify potential offset sites. The assumption is that companies prefer to consult already completed schemes that rank sites rather than conduct the conservation planning themselves. If the companies must conduct the conservation planning – and incur the costs of this planning – then they may be less likely to offset in the first place.

## Utility and discussion

The 40 schemes represent international and national-level approaches, and tend to be either area-based (Table 2, Additional file 1; two-thirds of the schemes; *e.g*., wetlands of international importance [Ramsar]) or taxa-based (Table 3, Additional file 2; 20%; *e.g*., IUCN red-listed species). A subset (15%) is a combination: they identify valuable areas based on their importance to particular taxa (reported in Table 2, Additional file 1; *e.g*., centers of plant diversity). About 10% of the schemes are essentially compilations of designations made by others (reported in  Table 3, Additional file 2 and Table 2, Additional file 1, depending on their focus; e.g., world database on protected areas).

The schemes share overlap in the criteria for inclusion. For example, a review of the eight best-known, scientifically-based schemes (identified by † in Table 2, Additional file 1; [10]) found that a few criteria were shared by most of the schemes, while other criteria arose only in a few (or none) of the schemes, in order of occurrence: intrinsic value of nature/wildlife (8 of 8 schemes); functionality (6); efficiency (6); international recognition and cooperation (4); representation (3); sustainable development (2); engaging local stakeholders (1); and utilitarian or sustainable use of wildlife (0). Endemism emerged as the most-often cited scientific criterion. Intactness was second.

Most of the earth's surface (79%) appears in at least one of the nine leading terrestrial-based schemes (identified by * in Table 2, Additional file 1), but it seems unlikely that business is going to restrict itself to the remaining 21%. Thus, companies need information at a spatial scale comparable to the impacts of their individual operations, *i.e*., at the site-level. Unfortunately, many schemes are defined over such large areas – hotspots, for example, cover entire countries – that few if any companies will chose a policy to stay out of hotspots altogether. This may explain in part why "a number of authors have pointed out that global conservation prioritization has had little success in informing actual conservation implementation" [11].

Coarse resolution also obscures variation in conservation value within a given priority area, *e.g*., within a hotspot, certain habitat types (such as wetlands or breeding sites) will have greater conservation value than others. Position within a landscape, such as proximity to wildlife corridors, creates further variability. However, the database (Table 3, Additional file 2 and Table 2, Additional file 1) reveals that only a few schemes operate at a resolution that can provide guidance at the site level. But even among schemes that identify site-level priorities, almost none (10%) rank values on a continuous scale. The vast majority of the schemes are dichotomous: a few ecosystems are identified as a priority while most other areas are left off the list.

Likewise, if a scheme does not assess threat then it is impossible to determine if an offset would provide any new benefits. If a site is not threatened then conservation actions are unnecessary (at least to prevent loss in the short term) and an offset would not provide an additional conservation gain. Unfortunately, the majority (80%) of schemes that focus on individual sites avoid the issue of current threat levels. Those that deal with threat are generally the schemes focused on endangered species.

It is not surprising, nor is it meant to be a criticism, that existing schemes are not more suitable for offsets – the schemes were developed with other objectives in mind. (Most are associated with particular conservation organizations and play a large role in their planning and fund raising.) Nonetheless, existing schemes can inform business of the major conservation issues in the regions where they operate. Moreover, where schemes do offer site-level priorities, such as important plant and bird areas (Table 2, Additional file 1), companies can use the schemes to identify no-go areas of such importance that no further impact is acceptable.

Where priorities have not yet been identified at the appropriate spatial scale and resolution, companies should collaborate with stakeholders to conduct a regional prioritization exercise. For example, a company could engage in an exercise akin to The Nature Conservancy's (TNC) Ecoregional Planning Framework or World Wildlife Fund for Nature's (WWF) Ecoregion Conservation Process [12] to meet the combined requirements of prioritizing sites based on representation, threat and viability. For a species-level approach, priorities could be derived from a process like that of Conservation International's (CI) Key Biodiversity Areas or Wildlife Conservation Society's (WCS) Range-Wide Priority Setting.

A substantial literature exists for the selection of representative reserve systems [13-18], including improved algorithms that explicitly incorporate threat levels (*e.g*., [19,20]), which should aid the ordering of conservation priorities for biodiversity offsets. Irreplaceability scores may serve as a relative measure of conservation priority, thus assisting in determining equivalence, and areas identified by high values of irreplaceability (*e.g*., 95%–100%) should be considered no-go areas [21,22]. The relative vulnerability of sites can then be used to further inform prioritization, albeit as a static assessment [19]. Despite the potential bias inherent in such a process, stakeholder workshops can also be an effective means of initiating a prioritization exercise [23].

The Convention on Biological Diversity calls for all signatory nations to prepare National Biodiversity Strategy and Action Plans, and in some cases these documents may serve as a comprehensive conservation 'needs assessment' for the nation (*i.e*., if they include identification of essential and priority targets for conservation as well as the interventions needed to sustain them).

## Conclusion

Offsets help reduce the perpetual fragmentation of landscapes by allowing companies to aggregate their conservation contributions into large areas of high conservation value (*e.g*., through conservation banks, or through informal, opportunistic collaborations). Where clear conservation priorities have not already been worked out, one would want to conduct a regional prioritization exercise, (see *e.g*., for North Zululand [24] or the Cape Floristic Region [25,26]), using the integrated conservation planning techniques mentioned above [27,28]. When setting priorities, both representation and persistence should be key targets: *i.e*., representation of all species and the habitats they occupy, and persistence of both species and the ecological and evolutionary processes that allow biodiversity to persist over time. As Brooks et al. [11] point out: "drawing the lessons of global conservation prioritization down to a much finer scale is now the primary concern for conservation planning." This database, especially with links to the schemes' website and maps, is a first step in this process. Our database can be used as an introduction to the conservation issues relevant to a given region. It may also provide a convenient starting point for new prioritization exercises, identifying key actors and resources (*e.g*., data sources, experts, publications).

## Availability and requirements

Our aim is to be inclusive, and we will continue to update the database on our website . We hope that users will assist with additions and comments to ensure that we maintain a living, comprehensive database.

Project name: BNI database of conservation prioritization schemes

Project home page: 

Operating system: Platform independent

Programming language: Excel

Licence: None needed

Any restrictions to use by non-academics: None

## Competing interests

The author(s) declares that there are no competing interests.

## Authors' contributions

All author(s) contributed equally, read and approved the final manuscript.

## Supplementary Material

Additional file 1 blundell burkey table. Click here for file

Additional File 2 Schemes that aim to prioritize species based on their conservation value Click here for file
