# Supplementary material for: A database of schemes that prioritize sites and species based on their conservation value: focusing business on biodiversity
Source: BMC Ecol. 2007 Sep 27;7:10. doi: 10.1186/1472-6785-7-10 (PMC2186304; doi:10.1186/1472-6785-7-10)
Supplement: Additional file 1 — blundell burkey table. [file 1472-6785-7-10-S1.pdf]

Table 2. Schemes that aim to prioritize sites/habitats/regions based on their conservation value

Scheme	Approach objective	# of cat.	Scale	Criteria	Types of activities acceptable
Italian wilderness areas<br>@	Try to maintain areas where human impact is low and traditional.	2	0 – 2	Areas where human impact is low.	Restricted, controlled use of resources, such as wood cutting, pasturage, and hunting rights. Where forest exploitation is permitted, it will be operated only with traditional methods of transport, with pack mules and horses.
Wetlands of International Importance—Ramsar Sites<br>@	The conservation and wise use of wetlands primarily to provide habitat for water birds	2	-2 – 4	Each country must list > 1 Wetland of International Importance based on their significance to waterfowl in terms of ecology, botany, zoology, limnology or hydrology. More specifically: (i) representative, rare, or unique example of a natural wetland found within the appropriate biogeographic region; (ii) supports vulnerable, endangered, or critically endangered species or threatened ecological communities; (iii) supports species important for maintaining the biological diversity of a particular biogeographic region; (iv) supports species at a critical stage in their life cycles, or provides refuge during adverse conditions; (v) supports 20,000 or more water birds; (vi) supports 1% of the individuals in a population of one species or subspecies of water bird; (vii) supports an indigenous fish subspecies, life-history stages, species interactions and/or populations that are representative of wetland benefits and/or values and thereby contributes to global biological diversity; (viii) an important source of food for fishes, spawning grounds, nurseries and/or migration paths.	Conservation and wise use of all wetlands through local, regional and national actions and international cooperation

World Heritage Sites @	Facilitate the implementation of the Convention concerning the Protection of the World Cultural and Natural Heritage	2	-2 – 5	(i) represent a masterpiece of human creative genius; (ii) exhibit an important interchange of human values; (iii) bear exceptional testimony to a cultural tradition or to a civilization which is living or which has disappeared; (iv) be an outstanding example of a landscape that illustrates significant stage(s) in human history; (v) an outstanding example of a traditional human settlement, land-use, or sea-use that is representative of a culture or human interaction with the environment especially when it has become vulnerable under the impact of irreversible change; (vi) be associated with events or living traditions, with ideas, or with beliefs, with artistic and literary works of outstanding universal significance; (vii) contain superlative natural phenomena or areas of exceptional natural beauty and aesthetic importance; (viii) be outstanding examples representing major stages of earth's history, including the record of life, significant on-going geological processes in the development of landforms, or significant geomorphic or physiographic features; (ix) be outstanding examples representing significant on-going ecological and biological processes in evolution and development; (x) contain the most important and significant natural habitats for in-situ conservation of biological diversity, including those containing threatened species of outstanding universal value.	Legislative and regulatory measures at national and local levels should assure the survival of the property and its protection against development and change that might negatively impact the outstanding universal value, or the integrity and/or authenticity of the property. States Parties should also assure the full and effective implementation of such measures.
Biosphere Reserves @	Cultural and Natural Heritage sites in ecosystems promoting solutions to reconcile the conservation of biodiversity with its sustainable use	2	-2 – 4	200 sites designated to achieve a range of objectives, including research, monitoring, training and conservation. Sites are: representative of a major biogeographic region, including a gradation of human intervention in these systems; contain landscapes, ecosystems or species, that need to be conserved; provide an opportunity to explore and demonstrate approaches to sustainable development; an appropriate size to serve the three functions of biosphere reserves mentioned above; zoned with a legally constituted core area, devoted to long-term protection; a clearly identified buffer zone and an outer transition area.	Organized into three interrelated zones, known as the core area, the buffer zone and the transition area, and only the core area requires legal protection

High Conservation Value Forests<br>@	Identify forests of outstanding conservation value	2	2 – 4	(i) forest areas contain significant concentrations of biodiversity values (e.g. endemism, endangered species, refugia); (ii) contain significant large landscape level forests, where viable populations exist in natural patterns of distribution and abundance; (iii) contain rare, threatened or endangered ecosystems; (iv) provide basic services of nature in critical situations (e.g. watershed protection, erosion control); (v) fundamental to meeting basic needs of local communities (e.g. subsistence, health); (vi) critical to local communities' traditional cultural identity (areas of cultural, ecological, economic or religious significance identified in cooperation with such local communities).	Designating a forest (or part of a forest) as HCVF does not automatically preclude management operations such as timber harvesting. However, it does mean that management activities must be planned and implemented in a way that ensures that the values are maintained or enhanced.
World Database on Protected Areas<br>@	The most comprehensive dataset on protected areas worldwide	2	-3 – 6	A fully relational database containing information on the status, environment and management of individual protected areas.	Depends on the restrictions imposed by each country.
Endemic Bird Areas †*<br>@	Priority areas for biodiversity conservation, drawing upon the expertise of an international network of ornithologists	2	1 – 6	218 regions of the world where the distributions of two or more restricted-range species overlap	No specific restrictions.
World Mangrove Atlas<br>@	The first significant attempt to provide an overview of the distribution of mangroves	2	1 – 3	ISME, in collaboration with ITTO and WCMC, are mapping the world's mangroves using GIS.	No specific restrictions.
Key Biodiversity Areas<br>@	Globally important sites that are large enough or sufficiently interconnected to support viable populations of the species for which they are important.	2	1 – 3	Presence of species for which site-scale conservation is appropriate: (1) globally threatened species, (2) restricted-range species, (3) congregations of species that concentrate at particular sites during some stage in their life cycle, and (4) biome-restricted species assemblages.	No specific restrictions, but conservation is the objective.

Natura 2000 Special Protection Areas (Europe)<br>@	Areas meant to protect certain rare or vulnerable populations of birds and their habitat	2	-2 – 4	The most suitable territories, in size and number, for listed species and for regularly occurring migratory bird species.	Listed wildlife and their breeding/resting sites may not be impacted.
Natura 2000 Special Areas for Conservation (Europe)<br>@	Contribute toward ensuring biodiversity through the conservation of natural habitats and of wild fauna and flora	2	-2 – 3	Support rare, endangered or vulnerable natural habitats and species of plants or animals (other than birds).	No specific restrictions.
Last Great Places (Latin America, US, Caribbean, Pacific)<br>@	Each place harbors concentrations of rare species and excellent examples of endangered terrestrial or aquatic ecosystems. Many places also are critical migratory stopover points for birds or form migratory corridors for other animals.	2		Healthy, functioning ecosystems in the U.S., Latin America, Caribbean and the Pacific where the sites are based on: vulnerability, threats, and the ability to lessen those threats and sustain biodiversity	No specific restrictions.
California Wilderness Study Areas<br>@		2	-2 – 3	(i) Preserve the unique wild and natural features of these landscapes; (ii) protect a diverse array of ecosystems, plants, animals, geologic structures and hydrologic features that represent the natural splendor of California; (iii) protect and preserve historical and cultural archaeological sites associated with ancient Indian cultures and the settlement of California; (iv) protect and preserve areas that continue to be used by Indian Tribes for spiritual, cultural, or subsistence practices; (v) protect watersheds, including those that play an essential role in providing municipal and agricultural water supplies; (vi) provide opportunities for compatible outdoor recreation; (vii) retain and enhance opportunities for scientific research in pristine ecosystems; and (viii) promote the recovery of threatened and endangered species, including salmon and steelhead.	

Last of the Wild†* @	To direct conservation attention to those places throughout the world relatively free of impacts by human beings	2	2 – 5	568 sites that represent the 10 largest, 10% wildest areas within each biome.	No specific restrictions.
Alliance for Zero Extinction Sites † @	Pinpoint and conserve epicenters of imminent extinctions. To identify and conserve all sites holding the entire global population of one or more Critically Endangered (CR)or Endangered (EN) species	2	-2 – 4	An AZE site must meet all three criteria: (1)Endangerment – must contain at least one EN or CR species, as listed by IUCN; (2) Irreplaceability – it is the sole area where an EN or CR species occurs, or contains the overwhelmingly significant known resident population, or contains the overwhelmingly significant known population for one life history segment (e.g., breeding or wintering); (3) Discreteness —the area must have a definable boundary within which the character of habitats, biological communities, and/or management issues have more in common with each other than they do with those in adjacent areas.	No specific restrictions, although the purpose is conservation.
Range-wide priority setting † @	Saving a species means saving its populations in all significantly different ecological settings in which they occur	2	1 – 5	Geographic units defined by potential habitat and bioregion across the species' historic range. Several criteria are used to prioritize units including representation, significant ecological processes, species decline, habitat loss, large intact areas and level of threat.	No specific restrictions, although the purpose is conservation.
Coral Reefs of the World @	Identify coral reefs worldwide	2	-2 – 4	WCMC-UNEP are mapping all coral reefs using GIS.	No specific restrictions.

Important Bird Areas † @	Identify, monitor and protect a global network of IBAs for the conservation of the world's birds and other biodiversity	2	-2 – 5	(i) species of global conservation concern. The site is known or thought to hold, on a regular basis, significant numbers of a Globally Threatened Bird species, or other bird species of global conservation concern. (ii) Assemblage of restricted-range species. The site holds a significant component of the restricted-range bird species whose breeding distributions define an Endemic Bird Area (EBA). (iii) Assemblage of biome-restricted species. The site holds a significant component of the group of bird species whose distributions are largely or wholly confined to one biome. (iv) Congregations. The site holds, on a regular basis, more than threshold numbers of a congregatory water bird, seabird or terrestrial bird species, or to exceed thresholds set for migratory species at bottleneck sites.	They become travel destinations and targets for eco-tourism projects and scientific study. Governments and donor agencies recognise the value of IBAs, so these sites attract financial incentives or direct funding for sympathetic development and management
Important Plant Areas @	Framework for identifying the very best sites for plants and fungi	2	-2 – 5	(i) The site holds significant populations of species of global or regional concern IUCN global red lists; (ii) The site has exceptionally rich flora in a regional context in relation to its biogeographic zone; (iii) The site is an outstanding example of a habitat type of global or regional importance on Annex 1 of the Habitats Directive (and any equivalent habitat from the Bern Convention Resolution 4)	No specific restrictions.<br><br>IPAs are an important aspect of addressing the Global Strategy for Plant Conservation Target (b) Conserving plant diversity, with subtargets:<br>- at least 10% of each of the world's ecological regions effectively conserved;<br>- protection of 50% of the most important areas for plant diversity assured;<br>- at least 30% of production lands managed consistent with the conservation of plant diversity;<br>- 60 %of the world's threatened species conserved in situ.
Crisis ecoregions* @	A comprehensive response aimed at protecting not only species, but the variety of landscapes, ecological interactions, and evolutionary pressures that sustain biodiversity, generate ecosystem services, and evolve new species	4	2 – 5	Ecoregions in which biodiversity and ecological function are at greatest risk because of extensive habitat conversion and limited habitat protection	

Vavilov Centres @	Certain geographic regions are characterized by an extraordinary range of wild varieties that can be regarded as the wild counterparts of cultivated species		5 – 7	14 areas of genetic diversity of wild relatives of domestic crop plants. These centres represent only a fortieth of the world's land area, and all are in the developing world.	No specific restrictions.
Global River Basin Analysis @	An outline assessment of pressures and the relative global importance of river basins	∞	2 – 7	151 river basins mapped. The focus is on highly diverse and highly stressed river systems. Stress is a combination of the water resource vulnerability index and the absence of wilderness in a catchment.	No specific restrictions.
Intact Forest Landscapes* @	To provide comprehensive information on logging, mining, and other activities within and around frontier forests as, or before, it happens	2	4 – 6	Roadless areas of forest > 50,000 ha.	No specific restrictions.
Intact Forest Landscapes –	To give an accurate picture of the current status of remaining intact forest landscapes	2	4 – 6	(i) forest zone; (ii) large (>50,000 ha); (iii) contiguous mosaic of natural ecosystems which may or may not be of different types; (iv) not fragmented by infrastructure; (v) does not display signs of significant transformation caused by human activity; and (vi) has a natural fire regime.	No specific regulations.
@	Canada	2	4 – 6		No specific regulations.
@	Russia	2	4 – 6		No specific regulations.
@	Sweden	2	4 – 6		No specific regulations.

Global 200 ecoregions †* @	To promote the conservation of terrestrial, freshwater and marine ecosystems harboring globally important biodiversity and ecological processes	2	3 – 6	The 200 ecoregions with the greatest species richness; endemism; higher taxonomic uniqueness; extraordinary ecological or evolutionary phenomena and global rarity of the major habitat type.	No specific restrictions.
Hotspots †* @	To protect the most species per dollar invested	2	4 – 6	Areas characterized both by exceptional levels of plant endemism and by serious levels of habitat loss; *i.e.,* those that contain at least 1,500 species of vascular plants (> 0.5 percent of the world's total) as endemics, and has lost at least 70 percent of its original habitat.	No restrictions – Conservation International aims to reduce further habitat destruction
Heartland Selection @	To protect large African landscapes of exceptional wildlife and natural value	2	4 – 6	Large, cohesive conservation landscapes that are biologically important and have the scope to maintain healthy populations of wild species and natural processes well into the future. They also form a sizeable economic unit in which tourism or other natural resource-based activities can contribute significantly to the livelihoods of people living in the area.	No specific restrictions.
Major tropical wilderness areas †* @	To focus biodiversity assessments in areas where conservation prospects are less complicated by human activities	2	4 – 7	37 areas that represent 46% of the Earth's land surface, but are occupied by just 2.4% of the world's human population, they have > 70% original vegetation, cover > 10,000 km2, and contain <5 people per km2	No specific restrictions.
Centers of plant diversity* @	Areas that if conserved, would safeguard the greatest number of plant species; and to document the many benefits, economic and scientific, that their conservation would bring	2	3 – 6	(i) species-rich, even though the number of species present may not be accurately known; (ii) the area is known to contain a large number of endemic species; (iii) contains an important genepool of plants of value to humans or that are potentially useful; (iv) contains a diverse range of habitat types; (v) contains a significant proportion of species adapted to special edaphic conditions; and, (vi) is threatened or under imminent threat of large-scale devastation.	No specific restrictions.

Megadiverse Countries* @	The most important countries for biodiversity—comparable to the G8 economic powerhouses	2	5 – 6	Top 17 countries with two-thirds of the worlds' biodiversity: Bolivia, Brazil, China, Colombia, Costa Rica, Democratic, Republic of the Congo, Ecuador, India, Indonesia, Kenya, Madagascar, Malaysia, Mexico, Peru, Philippines, South, Africa, Venezuela.	No specific restrictions.
Large Marine Ecosystems @	Advancing new policies and actions for eliminating causes of transboundary environmental and resource-use practices leading to serious degradation of coastal environments, linked watersheds, and losses in biodiversity and food security from overexploitation in large marine ecosystems	2	5 – 6	64 large ecosystem units defined as: regions of ocean encompassing near-coastal areas from estuaries out to the boundary of continental shelves and the seaward margins of coastal current systems. They are relatively large regions > 200 000 km^2, characterised by distinct bathymetry, hydrography, productivity and trophically dependent populations. Yields >95% of the usable annual global biomass yield of fishes.	No specific restrictions.

Links are provided to the website (@) and map/list () of each scheme; # of categories describes the number of categories that the scheme uses to rank priorities; *Scale* refers to the range in scale, as expressed in exponents (base 10) of km^2 (e.g., 3 = 10^3 km^2, or 1,000 km^2), at which the scheme operates on the ground; Criteria and Types of activities acceptable are quoted directly from the schemes' literature. † represents schemes covered by [9] and * by [10].
