# Supplementary material for: A database of schemes that prioritize sites and species based on their conservation value: focusing business on biodiversity
Source: BMC Ecol. 2007 Sep 27;7:10. doi: 10.1186/1472-6785-7-10 (PMC2186304; doi:10.1186/1472-6785-7-10)
Supplement: Additional File 2 — Schemes that aim to prioritize species based on their conservation value [file 1472-6785-7-10-S2.doc]

Table 3. Schemes that aim to prioritize species based on their conservation value

| **Scheme** | **Approach objective** | **# of cat.** | **Criteria** | **Types of activities acceptable** |
| --- | --- | --- | --- | --- |
| Endangered Species Act (USA)  [@](http://endangered.fws.gov/wildlife.html) [🌎](http://ecos.fws.gov/tess_public/StateListing.do?state=all) | To conserve ecosystems upon which endangered species and threatened species depend | 3 | An “endangered” species is one that is in danger of extinction throughout all or a significant portion of its range. A “threatened” species is one that is likely to become endangered in the foreseeable future. | Restricted use. |
| Species at Risk Act (Canada)  [@](http://www.speciesatrisk.gc.ca/default_e.cfm) [🌎](http://www.speciesatrisk.gc.ca/map/default_e.cfm) | To reverse the destruction of habitat, along with genetic and reproductive isolation, environmental contamination, over harvesting and excessive trade, climate change, disease and the presence of invasive species that threaten wildlife | 6 | 487 plant and animal species at risk. The species are listed in five categories: Special Concern, Threatened, Endangered, Extirpated, and Extinct. Experts regularly assess the status of wildlife. | Restricted use. |
| Australia's Endangered Species  [@](http://www.deh.gov.au/biodiversity/threatened/index.html) [🌎](http://www.deh.gov.au/cgi-bin/sprat/public/publiclistchanges.pl) | Protect Australia’s wildlife, of which about 85% of flowering plants, 84% of mammals, more than 45% of birds, and 89% of inshore, temperate-zone fish are endemic | 4-7 | Endangered, critically endangered, conservation dependent and extinct in the wild, vulnerable and extinct for wildlife species. Endangered, critically endangered, and vulnerable for ecological communities. | Restricted use. |
| European Protected Species  [@](http://www.naturenet.net/law/habsregs.html) [🌎](http://www.naturenet.net/law/europe.html#EuropeanProtectedSpecies) | Conserve wildlife in need of strict protections | 2 | For species where >10% are found in Europe and are endangered. | Listed wildlife and their breeding/resting sites may not be impacted. |
| National Biodiversity Index  [@](http://www.biodiv.org/gbo/annex.asp?ann=1) [🌎](http://www.biodiv.org/gbo/annex.asp?ann=1) | The conservation of biodiversity, the sustainable use of its components, and the fair and equitable sharing of benefits arising from use of genetic resources | - | An index based on a country’s richness and endemism in four terrestrial vertebrate classes and vascular plants; vertebrates and plants are ranked equally. Index values range between 1.000 (Indonesia) and 0.000 (Greenland). The NBI includes some adjustment allowing for country size. Countries with land area less than 5,000 sq km are excluded. | No specific restrictions. |
| CITES Appendix I - III  [@](http://www.cites.org/eng/res/09/09-24R13.shtml) [🌎](http://www.cites.org/eng/disc/species.shtml) | Through international cooperation, reduce the threats on wildlife from international trade | 3 | Species are listed on three appendices (I: endangered; II: threatened (trade allowed); III: species of concern). Detailed criteria based on population size, species range, and extent of decline. | Appendix I - no trade; Appendix II - trade if legally obtained and non-detrimental to survival of sp in its role in the environment; Appendix III - trade if legally obtained |
| Red Listed Species  [@](http://www.iucn.org/webfiles/doc/SSC/RedList/redlistcatsenglish.pdf) [🌎](http://www.redlist.org/search/search-expert.php) | System for classifying species at high risk of global extinction | 8 | Species are divided into the following: Extinct; Extinct in the Wild; Critically Endangered; Endangered; Vulnerable; Near Threatened; Least Concern; and Data Deficient. Detailed criteria based on population size, species range, and extent of decline. | No restrictions due to IUCN listing, however, countries may use the list to determine restrictions on certain categories. |

Links are provided to the website (@) and map/list (🌎) of each scheme; *# of categories* describes the number of categories that the scheme uses to rank priorities; *Criteria* and *Types of activities acceptable* are quoted directly from the schemes’ literature.
